# Propagation Techniques and Agronomic Requirements for the Cultivation of Barbados Aloe (*Aloe vera* (L.) Burm. F.)—A Review

**DOI:** 10.3389/fpls.2016.01410

**Published:** 2016-09-23

**Authors:** Giuseppe Cristiano, Bernardo Murillo-Amador, Barbara De Lucia

**Affiliations:** ^1^Department of Agricultural and Environmental Sciences, University of Bari Aldo MoroBari, Italy; ^2^Centro de Investigaciones Biológicas del Noroeste, Instituto Politecnico NacionalLa Paz, Mexico

**Keywords:** abiotic stress tolerance, burn plant, *in vitro* propagation protocols, crop husbandry, leaf processing, leaf yield

## Abstract

Barbados aloe (*Aloe vera* (L.) Burm. F.) has traditionally been used for healing in natural medicine. However, aloe is now attracting great interest in the global market due to its bioactive chemicals which are extracted from the leaves and used in industrial preparations for pharmaceutical, cosmetic, and food products. Aloe originated from tropical and sub-tropical Africa, but it is also now cultivated in warm climatic areas of Asia, Europe, and America. In this review, the most important factors affecting aloe production are described. We focus on propagation techniques, sustainable agronomic practices and efficient post harvesting and processing systems.

## Introduction

“Four vegetables are indispensable for the well being of man: wheat, grapes, olives and aloe. The first nourishes him, the second he refreshes the spirit, the third brings him harmony and the fourth cures him”

Christopher Columbus (1451-1506).

Aloe (*Aloe vera* (L.) Burm. F., Barbados aloe, *Xanthorrhoeaceae*, sub-family *Asphodeloideae*) is one of the most economically important medicinal crops worldwide with a significant bio-cultural value (Grace et al., 2009; Grace, 2011) and has a long history. Traditionally used for healing in natural medicine, for the last 20 years aloe has been at the center of a renewed interest in the global market due to its therapeutic and nutritive substances extracted from leaves, used in commercial preparations for pharmaceutical, cosmetic or alimentary uses and as a fresh food (Gutterman and Chauser-Volfson, 2007). The leaf mesophyll, commonly known as aloe gel, is subject to industrial processing to obtain derivatives.

Among its most representative organic biomolecules, aloe gel contains soluble sugars, anthraquinones, β-polysaccharides, amino acids, vitamins, glycoproteins, and enzymes (Chun-hui et al., 2007; Ahlawat and Khatkar, 2011; Chang et al., 2011; Lucini et al., 2015), with various biological properties such as antiviral, antibacterial, antifungal, anticancer, anti-inflammatory, wound healing, and many other characteristics that have prompted an increase in industrial and commercial production globally (Boudreau and Beland, 2006).

The succulent leaf tissue has an estimated annual market of $13 billion (Grace et al., 2015) which is likely to increase by 35–40% within the next 5 years. The USA dominates the market (65%), while India and China have a share of 10% each one (Biswas, 2010).

In addition to the demand from the pharmaceutical, cosmetics and processing industry, aloe has also been marketed in Europe and the USA through farmers' markets and e-commerce.

This global demand cannot be met solely by the wild harvesting of leaves aloe, which are indigenous to subtropical Africa (Van Jaarsveld, 1989; Van Wyk and Smith, 1996). Consequently, small and average-sized farms could exploit the increasing market demand worldwide, and invest in non-traditional crops, such as aloe (Liontakis and Tzouramani, 2016). Kenyan farmers see aloe cultivation as a strategy for reducing the risks linked to the fragile dependency on spontaneous aloe. This appears to have a greater potential to achieve the sustainable management of natural resources and to reduce poverty (Belmin et al., 2013). Since 2006, the Ethiopian government has recognized that the aloe plant is a source of income that integrates the diversification strategies for pastoral and agro-pastoral communities (Dirbaba, 2014).

Currently aloe is grown openly in the field in Kenya, Tanzania, Ethiopia, and Nigeria, with products exported to Europe and Asia (Oldfield, 2004); in south western Australia, China, India, Pakistan and Israel, Caribbean islands, southern USA, Mexico, and Paraguay. In Mediterranean climates (Spain, Greece, and southern Italy), it is cultivated both openly in the field and in cold greenhouses (De Lucia and Lucini, 2013).

Aloe can also be cultivated in some arid and semi-arid areas of the globe due to its eco-physiological characteristics: drought resistance, succulence, and a CAM trait.

Over the last decade, several scientific papers have dealt with the medicinal, pharmaceutical and nutritional properties of *A. vera*. This review brings together multiplication protocols, sustainable agronomic practices, efficient post harvesting and processing systems. Plant tissue culture techniques offer mass-production of consistently healthy planting material of a standardized quality. The most suitable agronomic management strategies can increase productivity, even surpassing that of species traditionally grown under the same conditions. Finally, suitable harvesting and post-harvesting conditions and appropriate processing methods can prevent the loss of bioactive chemical substances of the product.

## Propagation techniques

### Conventional *in vivo* propagation

The conventional propagation is not able to fulfill the increasing market demand for aloe, which calls for healthy, homogeneous, true-to-type, and available year-round plant material. Following, we provide an overview about the advantages and disadvantages of these propagation methods.

#### Propagation by seed

**Figure 1:**
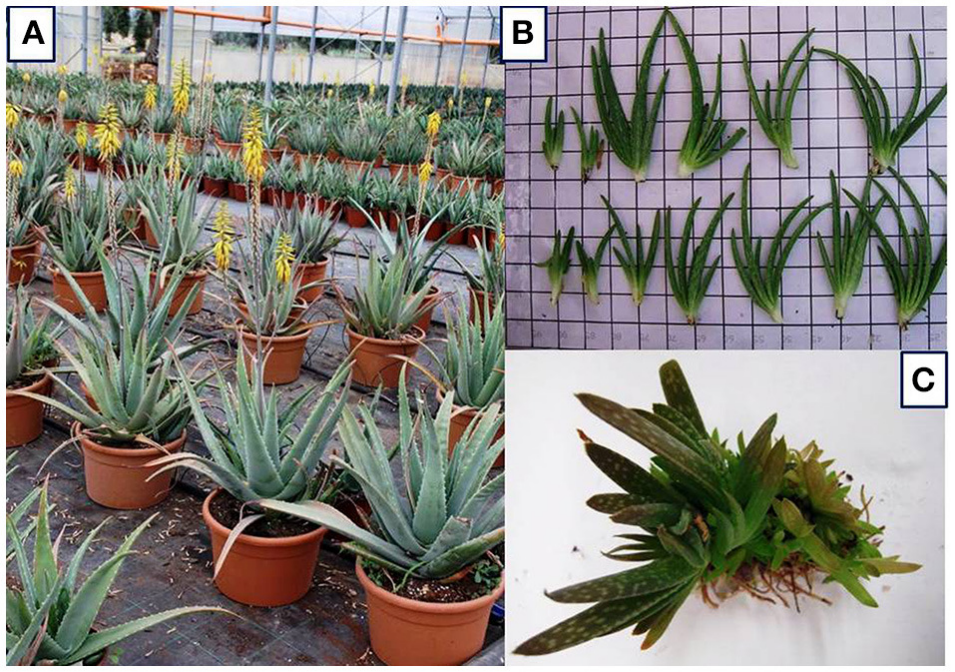
**(A)** *A*. *vera* (L.) Burm. F. plants with basal rosettes of leaves and inflorescence. Photo taken by De Lucia. **(B)** Suckers as *in vivo* planting materials. Photo taken by Cristiano. **(C)** *In vitro* culture: micropropagated shoots. Photo taken by Cardarelli.

The flower stalk starts from the center of the basal leaves with yellow flowers (Figure 1A); the fruit has a triangular shape and contains several seeds (Silva et al., 2010). Different drawbacks must be faced with the propagation by seed.

*A. vera* is generally self-incompatible, seeds are only fertile if they derive from cross pollination (Botes et al., 2009); this involves a high heterogeneity of the seed population (Ghate et al., 2002). Maintaining the species or varieties of aloe using sexual reproduction is difficult because the natural hybridization of this species is very frequent (Granados-Sánchez and Castañeda-Pérez, 1998). Nayanakantha et al. (2010a) and Alagukannan and Ganesh (2016) have revealed the existence of substantial genetic diversity among the Indian accessions for yield and quality parameters.

Seedlessness is another critical aspect (Gupta et al., 2015): a study by Sharma et al. (2011) suggests that both environmental and genetic factors are responsible for the anomalous chromosomal behavior that results in seedlessness.

Low percentage (0–25%) of seed germination is scored under *in vivo* condition. When germination is carried out *in vitro*, the seed germination percentage can reach 60–70% (Das et al., 2016).

In any case, plants raised by seed propagation take a long gestation period before attain the maturity: from 3 (Pushpan, 2012) to 4 years (Eshun and He, 2004) to attain harvestable size and it has a life span of about 12 years.

Therefore, commercial nurseries prefer to propagate *A. vera* agamically.

### Suckers and rhizome cuttings

Traditionally, *A. vera* is propagated using suckers (lateral shoots, Figure 1B) and rhizome cuttings as planting materials. In conventional production systems, each mother plant produces three to four suckers throughout the growing season (Pedroza-Sandoval and Gómez-Lorence, 2006; Smith and Van Wyk, 2009; Saggoo and Kaur, 2010; Gantait et al., 2014).

To prevent diseases, before transplantation in the field, selected suckers should be subjected to a slight moisture stress, under shade conditions (about 5–10 days). This then promotes root suberisation, which speeds up their adaptation and stimulates root growth when planting (Murillo-Amador et al., 2007). Crop is ready to harvest after 18 months of “sowing” (Das and Chattopadhay, 2004; Rajeswari et al., 2012).

Using rhizome to reproduce aloe on large scale (Rajeswari et al., 2012) is impractical due to the time required to have a completely developed plant (average 2 years), and to exposure to phytopathogens as a result of the cuts (Pedroza-Sandoval and Gómez-Lorence, 2006; Gantait et al., 2014).

Therefore, the high probability of damaging the mother plant and producing infected seedlings, the seasonality and the limited number of suckers along with the limited availability of raw material with high quality and slow vegetative growth are therefore limiting factors for a large-scale commercial production of young plants.

### *In vitro* propagation

To overcome the various difficulties of *in vivo* propagation, micropropagation can be exploited to obtain a large-scale production of uniform and healthy plantlets, which can be produced throughout the year (Gantait et al., 2014). *In vitro* propagation can be pursued through axillary bud stimulation and through *de novo in vitro* organogenesis (Rathore et al., 2011a,b; Gupta et al., 2014; Kanwar et al., 2015). In order to achieve a satisfactory production of healthy and standardized plantlets, the various stages of the *in vitro* cloning of *A. vera* need to be optimized.

#### Adventitious shoot regeneration

**Figure 2:**
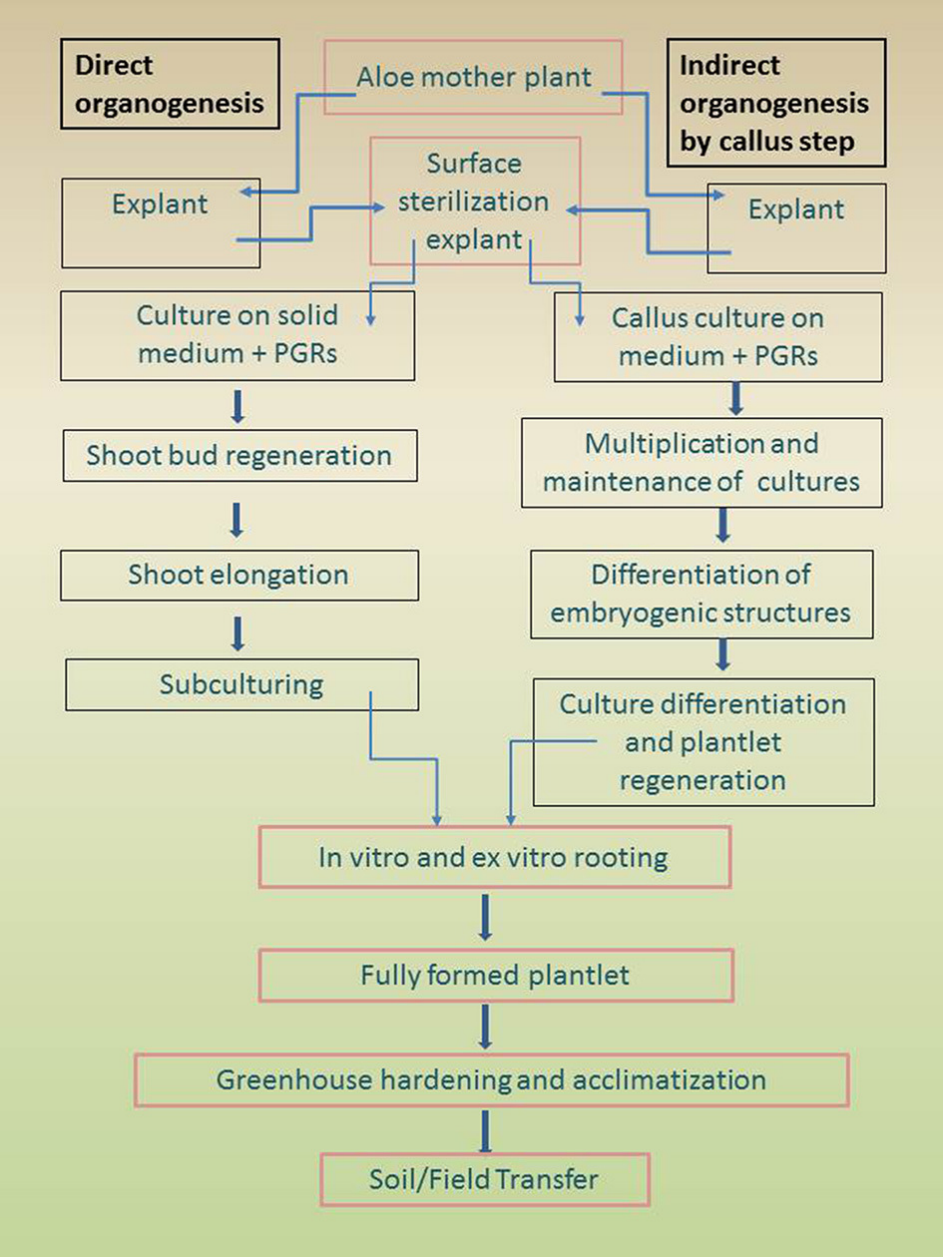
**Outline showing summary of tissue culture processes: direct and indirect organogenesis for aloe plantlet regeneration**.

Adventitious aloe shoots can be obtained through direct and indirect organogenesis (Figure 2).

During the direct pathway, the formation of a meristem proceeds without intermediate proliferation of undifferentiated callus tissue (Magyar-Tábori et al., 2010).

Direct shoot regeneration in *A. vera*, was achieved from leaf explants after 4–6 weeks of culture on MS medium (Murashige and Skoog, 1962) supplemented with 2.0 mg L^−1^ BA, 0.5 mg L^−1^ NAA, and 40 mg L^−1^ ADS (Sahoo and Rout, 2014). Rapid and large-scale indirect adventitious shoot regeneration was obtained from calli derived from the leaf base (Castorena-Sanchez et al., 1988; Lee et al., 2011), the leaf segments (Gupta et al., 2014; Kumari and Naseem, 2015), the leaf without sheaths (Abdi et al., 2013), the inflorescence axis (Rathore et al., 2011b), the stem segments (Gui et al., 1990), the stem discs (Saggoo and Kaur, 2010), and the rhizomatous stems (Roy and Sarkar, 1991). Seeds (Garro-Monge et al., 2008) and meristems (Abrie and Van Staden, 2001) have also been used as explants for callus induction.

Sterilizing the plant material is the most important step in the tissue culture protocol. Sterilization involves eliminating microbial contaminants from the surface and interior of plant material, thus giving a greater chance of survival *in vitro*.

Saggoo and Kaur (2010) and Rathore et al. (2011b) showed that a pre-treatment with a combination with Bavistin and streptomycin and subsequent surface sterilization, prevented fungal and bacterial growth in cultures.

The plant material can be disinfected with mercuric chloride (Liao et al., 2004; Ahmed et al., 2007; Kumar et al., 2011) which is now seldom used as it is dangerous (Kaviani, 2015), and sodium hypochlorite (Natali et al., 1990; Nayanakantha et al., 2010b; Adelberg and Naylor-Adelberg, 2012). Browning decreases the explant survival and leads to failures in shoot regeneration (Roy and Sarkar, 1991). A suitable carbon source and antioxidants (ascorbic and citric acids) need to be added to avoid excessive phenolic secretion in the culture medium (Das and Srivastav, 2015). To eliminate blacking and browning, PVP can be used (Roy and Sarkar, 1991; Liao et al., 2004; Rathore et al., 2011b). Nayanakantha et al. (2010b) reported that a medium containing 1.0 g L^−1^ PVP + 10 mg L^−1^ citric acid + 500 mg L^−1^ AC resulted in blocking the browning of the medium due to tissue phenols release.

The initiation of regenerative callus culture is dependent on the nature and concentration of PGRs: auxins (IAA, IBA, NAA, or 2,4-D) are also often added to the culture medium to regulate morphogenesis, alone or in combination with cytokinins.

Gui et al. (1990), in stem segments, initiated callus cultures of *A. vera* on MS medium with NAA alone or with 2.0 mg L^−1^ Zeatin; Roy and Sarkar (1991) with a combination of 1.0 mg L^−1^ 2,4-D and 0.2 mg L^−1^ Kin.

The soft base of young inflorescence axis as explants, after 30–35 days, produced callus on 0.8% agar gelled MS medium supplemented with 6.0 mg L^−1^ 2,4-D and 100 mg L^−1^AC (Rathore et al., 2011b). The Authors suggested that to get regenerative globular structures a decreased PGRs concentration is necessary (MS medium with 1.0 mg L^−1^ 2,4-D). NAA only has been proved to be essential for optimum callus induction from leaf segment explants (Abdi et al., 2013).

In other studies, callus developed in the MS medium supplemented with BAP, obtaining the maximum percentage (100%) of culture within the minimum time required: 9.6 days (Gupta et al., 2014).

Rathore et al. (2011b) found that more than three to four passages lead to hyperhydricity of cultures probably caused by high concentrations of cytokinins: for this reason, once meristematic activity is achieved, the concentration of PGR needs to be decreased. The regeneration from callus culture in adventitious shoot occurred with BAP alone (Rathore et al., 2011b) and BAP in combination with NAA: Gupta et al. (2014) observed the best and most rapid microshoot formation on MS supplemented with 2.0 mg L^−1^ BAP + 0.5 mg L^−1^ NAA, which yielded the highest number (75) of regenerated shoots, with an average length of 4.0 cm.

Regenerated shoots were cultured on MS basal medium fortified with 0.5 mg L^−1^ NAA for adventitious rooting from the 10th day of culture, and 90% of root formation took place within a period of 3–4 weeks for maximum callus induction. The highest number of roots per shoot was 4.8, with an average length of 3.5 cm (Gupta et al., 2014).

For large or mass-scale production, the efficiency of the propagation methods is important, but perhaps even more important is the genetic stability. Rathore et al. (2011a) revealed a somaclonal variation of more than 70% in plants propagated by indirect organogenesis from the base of the inflorescence axis. This genetic instability, mainly in callus-derived plants, is a negative factor for the nursery industry, which uses axillary branching technique and to produce true-to-type clones of the aloe mother plants.

Various types of plant organs are used to promote adventitious shoot regeneration: when shoots are developed directly from leaf explants, i.e., without intervening callus phase, this is referred to as direct organogenesis.

#### Propagation through axillary branching

Axillary branching miniaturizes the natural process of branching. The cytokinins, supplemented to the culture medium, overcome apical dominance releasing lateral buds from dormancy (George et al., 2008) and BA and BAP (same compound named differently according to Teixeira da Silva, 2012) are the synthetic cytokinins commonly used. By this way, elite plant material can be efficiently produced with low or no somaclonal variation (Nayanakantha et al., 2010a). The following different steps should be faced to reach a satisfactory protocol to obtain virus-free plants.

##### Culture establishment

Various types of explants are used to get axillary branching: meristem in order to obtain virus-free aloe (Campestrini et al., 2006; Das et al., 2010; Rizwan et al., 2014), shoot tips (Meyer and Van Staden, 1991; Aggarwal and Barna, 2004; Ahmed et al., 2007; Supe, 2007; Hashemabadi and Kaviani, 2008; Zakia et al., 2013; Amoo et al., 2014; Molsaghi et al., 2014), axillary shoot segments (Singh et al., 2009; Shekhawat et al., 2013), shoot bud (Kanwar et al., 2015), apical buds (de Oliveira et al., 2009; Gupta et al., 2014), buds from underground stems (Liao et al., 2004), stem nodes (Singh and Sood, 2009), rhizomatous stems (Gantait et al., 2010, 2011), and suckers (Nayanakantha et al., 2010b).

*In vitro* culture establishment is the first stage of the protocol. It is influenced by the physiological state of the source plant and its age as well as the season of explant collection. The treatments for the explants are critical as these determine their growth and behavior in culture (Singh et al., 2009).

Aggarwal and Barna (2004) developed a cost effective and efficient method of micropropagation through axillary branching: shoot tip explants (2–3 cm), inoculated on MS medium containing 0.2 mg L^−1^ BA and 0.2 mg L^−1^ IBA for shoot initiation, showed signs of proliferation after 2 weeks. New buds appeared from the axils of leaves and developed into shoots by the 4th week of culture.

Table 1 shows that the percentage of success for the initiation phase can reach 93% (Gantait et al., 2011) or 100 % (Shekhawat et al., 2013).

**Table 1:** **Effect of different cytokinin concentrations on ***in vitro*** success percentage of initiation phase (S.I.) and multiple shoot formation (M.S.F.) in ***A. vera*****.

**References**	Explant (^*^)	**MS** + **Growth regulators Cytokinin (mg L**^−1^**)**			**S.I. (%)**	**M.S.F**.	**Shoot length (cm)**	**Time (weeks n)**
		BA (^**^)	BAP (^**^)	**KIN**				
de Oliveira et al., 2009	Ab	0	2.0	0	Dnp	5.3	Dnp	Dnp
Singh et al., 2009	Ass	0	3.0	0	Dnp	10.3	2.5	Dnp
Gantait et al., 2011	Rs	0	2.5	0	93	9.7	3.8	4
Shekhawat et al., 2013	Ass	0	3.0	0	100	10.0	Dnp	4

* *Ab, Apical bud; Ass, Axillary shoot segments; Rs, Rhizomatous stem; Dnp, Data not presented*.

** *Teixeira da Silva (2012) reports that these two different names refer to the same cytokinin*.

##### In vitro multiplication

Various basal media, such as B5 (Gamborg et al., 1968) and SH (Schenk and Hildebrandt, 1972) have been employed for micropropagation, but the most widely used culture medium is MS (Murashige and Skoog, 1962). The most used growth regulator to induce shoot initiation and multiplication was the cytokinin BAP or BA. Although Teixeira da Silva (2012) clearly explains that these two different names refer to the same cytokinin, in our review we prefer to maintain the original nomenclature used by the authors.

Shekhawat et al. (2013), Singh et al. (2009), de Oliveira et al. (2009), and Gantait et al. (2011), used BAP alone, at concentrations respectively of 3.0, 2.5, 2.0, and 0.7 mg L^−1^, with a shoot multiplication rate between 10 and 5.3.

Some researchers reported that a combination of PGRs improved micropropagation: Natali et al. (1990) reported multiplied shoots on MS supplemented with 0.5 mg L^−1^ Kin and 0.2 mg L^−1^ of 2,4 D; Kanwar et al. (2015) obtained maximum average number of shoots (5.5) per explant and maximum average shoot length (3.3 cm) on MS medium supplemented with 2.2 mg L^−1^ BA and 1.6 mg L^−1^ Kin.

Campestrini et al. (2006), Zakia et al. (2013), and Gupta et al. (2014) demonstrated that the best combination for the growth of aloe microshoots was MS medium supplemented with BAP and NAA. Supe (2007), Molsaghi et al. (2014), and Rizwan et al. (2014) reported that the best multiplication of shoots was obtained on medium containing BAP + IAA. Das et al. (2010) cultured aloe plantlets on medium containing BAP and IBA which produced the highest number of shoot buds (22.0) and enhanced bud proliferation within 1 to 2 weeks after the first subculture. Ahmed et al. (2007) reported 15.4 shoots per explant in MS medium containing a combination of 2.0 mg L^−1^ BA, 0.5 mg L^−1^ Kin and 0.2 mg L^−1^ NAA. Hashemabadi and Kaviani (2008) reported the maximum number of shoots (around 9.7 per explant) in MS medium supplemented with 0.5 mg L^−1^ BA and 0.5 mg L^−1^ NAA.

Regarding the shoot multiplication rate, Singh et al. (2009), Gantait et al. (2011) and Abdi et al. (2013) achieved a rate of more than 9, which was obtained 4 weeks after establishment, respectively with BAP 3.0, BAP 2.5, and BA 4.0 mg L^−1^. Variations in cytokinin level (BAP 2.0 and BA 10 mg L^−1^) reduced shoot proliferation (de Oliveira et al., 2009; Kanwar et al., 2015). Adelberg and Naylor-Adelberg (2012) reported that 1.3 mg L^−1^ BA in liquid medium is optimal for multiplication rate. Several studies (Liao et al., 2004; Ahmed et al., 2007; Supe, 2007; Gupta et al., 2014; Molsaghi et al., 2014; Rizwan et al., 2014) reported that improved *in vitro* multiplication can be scored when the cytokinins are associated with the auxins (multiplication rate from 1:9.5 to 1: 58 respectively on MS medium with BAP 1.5 mg L^−1^, NAA 0.5 mg L^−1^ and on the same medium with BAP 4.0 mg L^−1^ and IAA 1.0 mg L^−1^ (Table 2, Figure 1C).

**Table 2:** **Effect of different concentrations and combinations of BA, BAP, KIN, and IBA, NAA, IAA on success percentage of initiation phase (S.I.) and multiple shoot formation (M.S.F.) in ***A. vera*****.

**References**	Explant (^*^)	**MS** + **Growth regulators (mg L**^−1^**)**						**S.I. (%)**	**M.S.F**.	**Shoot length (cm)**	**Time (weeks n)**
		**Cytokinin**			**Auxin**						
		BA (^**^)	BAP (^**^)	**KIN**	**IBA**	**NAA**	**IAA**				
Liao et al., 2004	Bus	2	0	0	0.2	0	0	Dnp	15.0	Dnp	4
Aggarwal and Barna, 2004	St	1	0	0	0.2	0	0	100	3.3	Dnp	4
Campestrini et al., 2006	M	0	1.5	0	0	0.7	0	Dnp	8.0	Dnp	4
Ahmed et al., 2007	St	2	0	0.5	0	0.2	0	99	15.4	1.5	5
Supe, 2007	St	0	4	0	0	0	1	Dnp	22.4	Dnp	4
Hashemabadi and Kaviani, 2008	St	0.5	0	0	0	0.5	0	100	9.7	3.5	4
Singh and Sood, 2009	Sn	2	0	0	2	0	2	Dnp	6.4	Dnp	4
Das et al., 2010	Sam	0	8	0	2	0	0	83	9.7	Dnp	4
Lee et al., 2013	Ms (^***^)	1	0	0	0	0.1	0	72	6.6	1.6	5
Zakia et al., 2013	St	0	0.5	0	0	0.5	0	Dnp	11.2	12.2	7
Gupta et al., 2014	Sn	0	1.5	0	0	0.5	0	100	9.5	3.7	4
Molsaghi et al., 2014	St	0	4	0	0	0	1	Dnp	58.0	6.0	4
Rizwan et al., 2014	Sam	0	3	0	0	0	1	100	27.7	Dnp	4
Kanwar et al., 2015	Sb	10	0	5.0	0	1.5	0	72	5.5	3.4	4

* *Bus, Bud from underground stem; Ms, Meristem segments; Sam, Shoot apical meristem; Sb, Shoot bud; Sn, Stem nodal; St, Shoot tip; Dnp, Data not presented*.

** *Teixeira da Silva (2012) reports that these two different names refer to the same cytokinin*.

*** *in MS + spermidine 50*.

Table 2 shows that the longest microshoot (12.2 cm) was obtained after 7 weeks on MS medium supplemented with 0.5 mg L^−1^ BAP + 0.5 mg L^−1^ NAA (Zakia et al., 2013).

Repeated subculturing of the micro-shoots has been suggested as a method to rejuvenate adult tissues (Kanwar et al., 2010). Gantait et al. (2010), noticed no difference in the number of shoots between the first and fifth subcultures, whereas Kanwar et al. (2015) obtained 22.7 shoots/explants after the sixth subculturing, with an average length of 9.0 cm, compared to the first with only 5.5 shoots/explant with an average length of 3.5 cm. Hashemabadi and Kaviani (2008) obtained 12 shoots/explants in the fourth subculturing. Kanwar et al. (2015) observed that the *in vitro* rooting response increased as a function of the *in vitro* culture cycle applied. At the sixth subculturing, the length root was 3.7 cm. The same authors, in another experiment, transferred explant as juvenile leaf, after every 3 days, into fresh shoot induction full strength MS medium supplemented with 0.04% AC and incubated it at 25 ± 2°C under 16 h photoperiod. They obtained a phenolic-free microshoot; AC provided the dark conditions required for the root initiation and growth and also adsorbed the substances presumed to be detrimental.

The automation of micropropagation in bioreactors on a liquid medium is one way of reducing propagation costs, as reported by Adelberg and Fári (2010). This is because the explant multiplies more rapidly, a better nutrient availability is established and once the microshoots are acclimatized under *in vivo* conditions, an improved growth could be observed compared to the traditional *ex vitro* plant material (Cardarelli et al., 2014). To override tissue hyperhydricity, Ziv (2005) and Tascan et al. (2010) proposed the addition of polyester fiber matter without mechanical ventilation.

##### In vitro rooting

Rooting is the last *in vitro* step before the plantlets are transferred to *ex vitro* conditions. Rooting success (Table 3) ranges from 80 (Ahmed et al., 2007) to 100% (Singh et al., 2009; Abdi et al., 2013; Molsaghi et al., 2014).

**Table 3:** **Effect of different concentrations and combinations of IAA, NAA, KIN, and BA on ***in vitro*** rooting in ***A. vera*****.

**References**	**MS** + **Growth regulators Auxin** + **Cytokinin (mg L**^−1^**)**				**Rooting success (%)**	**Root/shoot (n)**	**Root length (cm)**	**Time (week n.)**
	**IAA**	**NAA**	**Kin**	BA (^*^)				
Aggarwal and Barna, 2004	0	0	0	0	90	2.8	15.7	2.0
Ahmed et al., 2007	0	0.2	0	0	80	6.7	2.7	3.0
Supe, 2007	1	0	0	0	85	12	Dnp	4.0
Hashemabadi and Kaviani, 2008	0	0.5	0	0.5	Dnp	Dnp	Dnp	Dnp
Singh et al., 2009 (^**^)	0	0	0	0	100	Dnp	4.0	1.8
Gantait et al., 2011	0.5	0	0	0	97	3.3	3.7	3.0
Abdi et al., 2013 (^***^)	0	2	0	0	100	7.8	15.7	3.0
Gupta et al., 2014	0	0.5	0	0	95	4.8	3.5	4
Molsaghi et al., 2014	0.1	0	1	0	100	Dnp	9.8	1.8

* *Teixeira da Silva (2012) reports that BA and BAP refer to the same cytokinin*.

** *1/2 semi-solid MS medium*;

*** *B5 medium; Dnp, Data not presented*.

Aggarwal and Barna (2004) observed a number of roots per shoot (2.8) in MS medium without any PGR supplementation; Supe (2007) reported 12 roots/shoot. Hashemabadi and Kaviani (2008) reported that a dose of BA during multiplication phase of more than 0.5 mg L^−1^ reduces the rooting percentage of the shoot as various proteolytic enzymes are suppressed.

Subsequent protocols have exploited the use of auxins (Table 3): IAA alone (Supe, 2007; Gantait et al., 2011), and NAA alone (Ahmed et al., 2007; Abdi et al., 2013; Gupta et al., 2014).

The addition of AC in the culture medium improves the root number of micropropagated shoots (Abadi and Kaviani, 2010), and also ensures a better plant growth during acclimatization (Borgognone et al., 2013). Singh et al. (2009) proposed a protocol on *ex vitro* rooting. They found that when the *ex vitro* rooting of shoots and the hardening of plantlets are achieved in the greenhouse in a single step, the protocol takes a shorter time for plantlet production and is considerably cheaper.

##### Acclimatization

The success of a micro-propagation protocol depends on the *ex vitro* acclimatization phase of the micro-propagated plantlets, which is measured by the percentage survival. A 70% survival rate was achieved by Gupta et al. (2014).

This success has been positively correlated to the dimensions of the *ex vivo* microplants: medium (6–8 cm long bearing 4–5 leaves) and large (13–16 cm long bearing 5–7 leaves) sizes have been found to be significantly different from small (3–4 cm long bearing 2–3 leaves) microplants (de Oliveira et al., 2009).

Supe (2006) demonstrated the need for direct sunlight throughout the acclimatization of micropropagated aloe. Hosseini and Parsa (2007) used compost, loam, and clay with 1:2:2 ratios as a substrate for acclimatization, where the plants were covered with transparent plastic pots to retain humidity throughout the process.

More than 2000 well-acclimatized plants were produced over a period of 6 months from five explants (Meyer and Van Staden, 1991).

##### Notes on the agronomical performance of ex vitro plantlets

The composition of aloe gel depends upon the method of propagation. Pandhair et al. (2011) showed that total sugars, fructose, sucrose, starch and phenol content were higher in micropropagated leaf gel as compared to sucker-propagated plants. Addition of 20 mg L^−1^ tryptophan induced a 2.43-fold increase in aloin content in multiple shoots cultures (Sharma V. et al., 2013).

Lee et al. (2011) reported that aloe-emodin content was dramatically increased in the adventitious roots grown in B5 media to 20~40 fold higher than that in MS medium.

**Figure 3:**
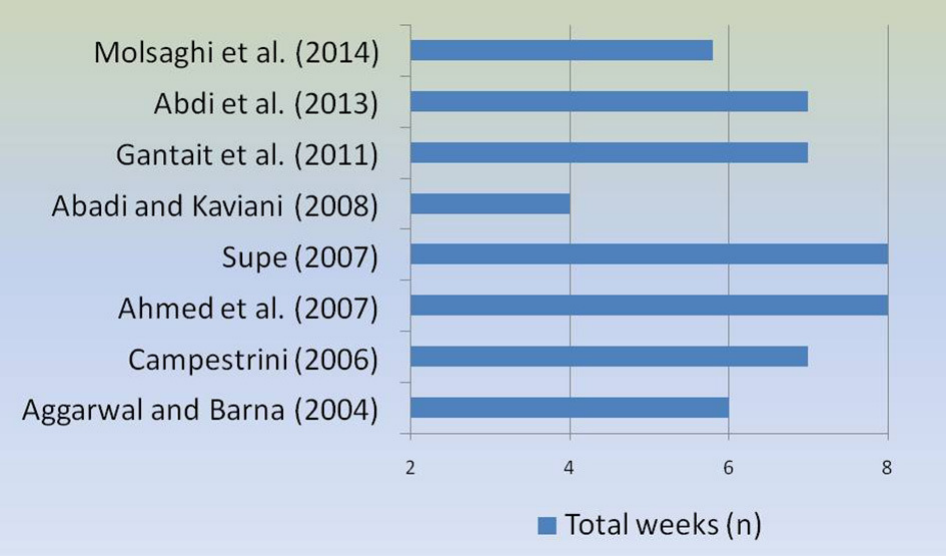
**Number of weeks from ***in vitro*** shooting to rooting in different protocols**.

The Figure 3 shows the average number of total weeks from *in vitro* shooting to rooting, in different researches.

**Figure 4:**
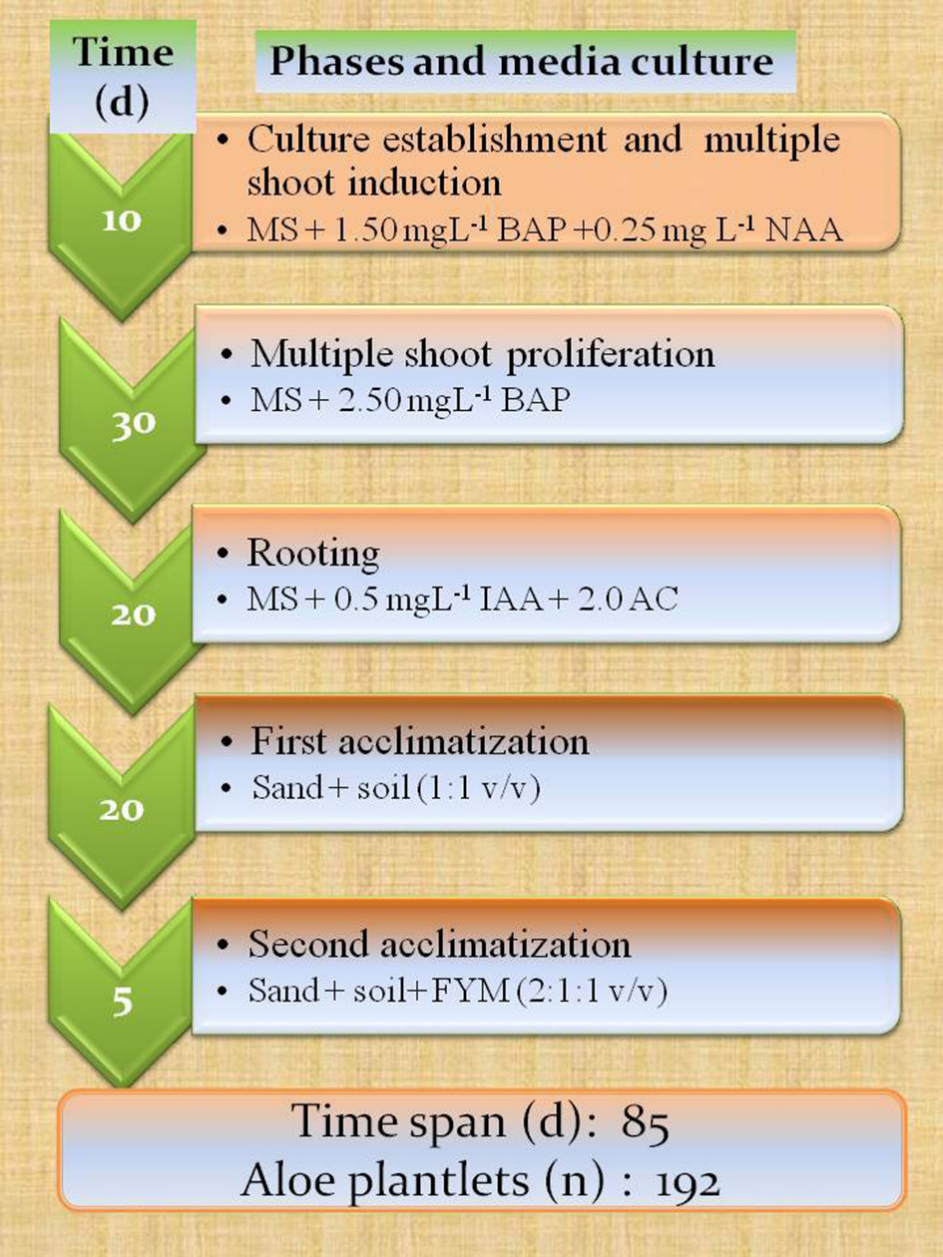
**Fast mass propagation calendar: from multiple shoot formation to acclimatization phases**. Results shown are adapted from Gantait et al. (2011). Teixeira da Silva (2012) reports that BA and BAP refer to the same cytokinin.

A more extended time reference, such as a very fast protocol, was proposed by Gantait et al. (2011), which enabled them to obtain 192 true to type plants from a single explant, with a rhizomatous stem, in a total period of 85 days from bud induction to the end of acclimation (Figure 4).

Singh et al. (2009) described a very cheap protocol on liquid substrate that starts from a single bud and produces 5000 plantlet in 180 days. Rathore et al. (2011b) transferred in fields under the environmental conditions 1900 tissue-cultured propagated plantlets.

The cost of a micropropagated plantlet ranges between 1.0 and 1.50 $ and is still too high for farmers in developing countries to purchase it on a large scale whereas the planting density is between 12,000 and 20,000 plants/ha.

## Climatic and soil requirements

Although McKeown (1987) describes aloe as a subtropical species that is intolerant of low temperatures, an experiment in the Golan Heights (Israel) in 1995 showed that it can reach commercial size with an absolute minimum temperature of −3°C and up to 2 months with a temperature of ≤ 4°C (Saks and Ish-Shalom-Gordon, 1995).

Irradiance significantly affects plant development (Pedroza-Sandoval and Gómez-Lorence, 2006). In the cultivation of young plants, Paez et al. (2000) in Chile showed a direct correlation between partial shade (30% full sunlight) and yield: with 27% more leaves than plants under full sunlight.

Aloe is grown in all kinds of soil with a high organic matter content (Kent, 1980). Good drainage of the soil profile must be guaranteed to prevent puddling, which could cause root asphyxia and the spread of root apparatus diseases such as tracheo-micosis by both *Fusarium oxysporum* and *Verticillum* sp. (Ayodele and Ilondu, 2008). The channeling of drainage water must also prevent the formation of surface puddles during the rainy season.

## Soil and soilless cultivation

### Soil cultivation

#### Transplant

**Figure 5:**
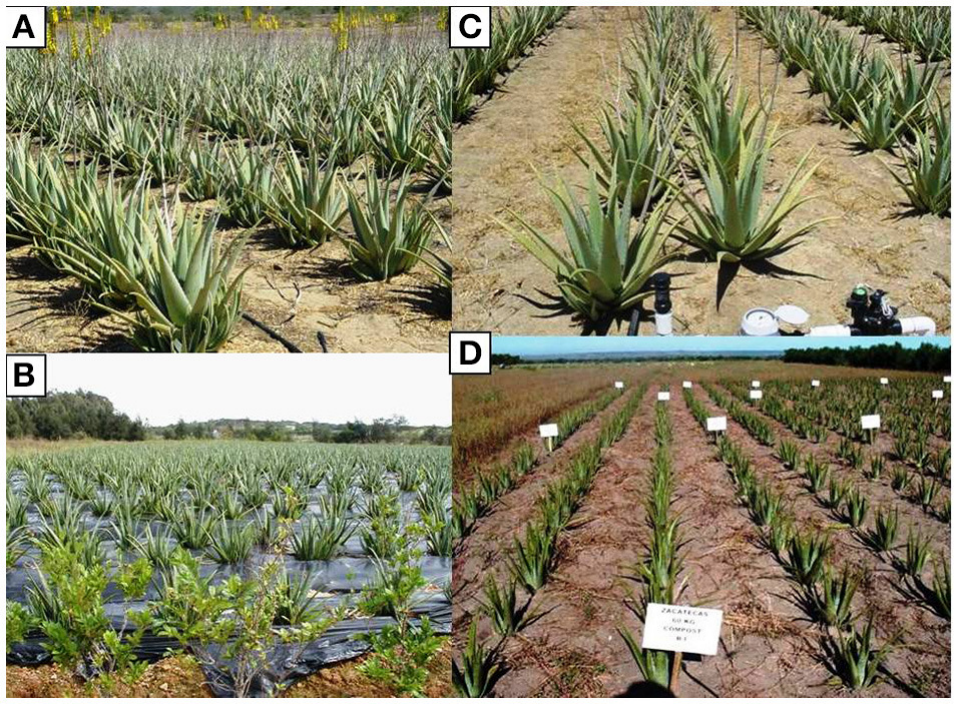
**(A)** Plantation densities as 20,000 plants ha^−1^ in the open field. **(B)** Mulching with black polyethylene film in the open field. **(C)** Drip irrigation system in open field cultivation. **(D)** Organic fertilization as manure compost. Photos taken by B. Murillo-Amador.

In open fields under irrigated conditions transplant is carried out throughout the year, except in winter (Rajeswari et al., 2012). Land preparation is limited to the surface to prevent erosion, which is common in recent plantations. Rajeswari et al. (2012), in West Bengal area, reports that suckers are planted in roughly 15 cm deep pits, 60 × 60 cm apart, which are dug at the time of planting. After planting, the soil around the root zone must be firmly pressed and proper drainage must be ensured to prevent water stagnation. Saha et al. (2005) have shown that the optimal layout is with 100 cm apart rows and plants every 50 cm in the rows, with 20,000 plants per hectare. Murillo-Amador et al. (2007) reported, in Mexico, higher leaves and gel yield using 25,000 plants ha^−1^, with 90 cm between rows and 45 cm among plants, with a double quincunx row arrangement (Figure 5A).

Hernández-Cruz et al. (2002) report that mulching the soil with a 100 μm thick black polyethylene film (Figure 5B) positively influences the gel content in the leaves.

#### Weed management

According to CONAZA (1994) in areas that are less than one hectare, the manual method is the most common way to eliminate weeds in aloe plantations, which is preferred by farmers because the use of machinery decreases soil porosity, aeration and drainage. Generally, two weed eliminations per year after the rainy season are enough but in some regions farmers maintain weeds before the winter season in order to conserve microclimate conditions around the plants and thus protect them from low temperatures (Santos, 1995). Also, some farmers manually remove some parts of the plants such as suckers (5–10 cm) and the inflorescences to enable the plants to acquire energy for growth and to increase yield.

For aloe plants under warmer climate conditions and a rainfed cultivation system, four weed controls are necessary, three during the rainy season and the fourth during the dry season. Chemical control of weeds is not permitted by aloe industrial regulations. However, if the weeds are not controlled, the yield decreases and some climbing weeds can bind the leaves and damage them (Rodríguez-Canto, 1992). Most aloe growers avoid using chemical pesticides and herbicides and some let geese into the fields to eat the insects that are uncovered by plowing (Gage, 1996). According to Pedroza-Sandoval and Gómez-Lorence (2006), the weeds are most common in Aloe cultivated under irrigation systems and the type of weeds depends on the agricultural region of each country. When the aloe plants are small it is possible to use machinery to eliminate weeds, however this is not recommended when plants are growing, because the machinery can damage the plants.

The best method is to eliminate weeds manually. In fact, Gage (1996) affirms that a plants must be planted, cultivated and harvested by hand. In cultivated areas greater than one hectare, there are generally various alternative methods to eliminate weeds. For example, in some regions of Mexico, the farmers introduce goats and lambs to consume the weeds, since these animals do not feed on aloe. Another way to eliminate weeds it is to use padded plastic, which preserves the soil humidity, and controls weeds, pests and pathogens in the soil. In addition, the use of industrial aloe leaf waste, known as bagasse, is commonly used as an organic cover to prevent weeds. Bagasse also preserves the soil humidity and organic matter is incorporated to the soil. Although the use of chemical products to control weeds is not permitted in aloe, Pedroza-Sandoval and Gómez-Lorence (2006) report the use of glyphosate (Round-up®), while PLM (2015) report the use of atrazine (Atranex® 50% SC) as active ingredients to control weeds in aloe.

#### Water management

CAM plants are characterized by the maintenance of a favorable water status through their ability to minimize transpiration by closing their stomata during the day and opening them at night when the vapor pressure deficit is low (Caird et al., 2007). Water loss is reduced by the nocturnal CO_2_ uptake with an efficient synthesis of sugars and osmolytes which allow water retention (Borland et al., 2009). Another common feature of CAM plants is succulence, characterized by cells with large vacuoles, called hydrenchyma; the internal water reserve in fleshy leaves enable aloe to survive dry seasons or cyclical droughts (Newton, 2004). The plant requires periodic rainfall or irrigation to replenish depleted reserves.

In open field cultivation, in arid and semi-arid climates, it is necessary to irrigate to ensure both continuous growth and gel production. An excessive volume of water is thus avoided, which could damage the collar and cause rotting.

A water deficit negatively influences the plant growth through a substantial reduction in the number of leaves and suckers (Genet and van Schooten, 1992). Winter et al. (2005) found that efficiency of water use, in potted aloe, can reach 54g H_2_O g^−1^ dry matter.

Rodríguez-García et al. (2007) found that low soil water content reduced the fresh weight, number of leaves and growth rate of the plants.

Silva et al. (2010) found that the water requirement of 15% of the reference evapotranspiration (ET_0_) led to the maximum biomass productivity.

Murillo-Amador et al. (2007) evaluated the effect of one irrigation volume (20 mm per hour), different times (1.5 and 3 h) and two intervals (once and twice per week) in the production of leaf biomass and gel in an arid zone of Mexico (Figure 5C). Their results showed that the production of leaves and gel increased when 20 mm per hour were applied for 3 h, once per week.

#### Nutrient management

Fertility in agronomic management is one strategy to boost the aloe yield, however to reduce production costs aloe fields are often not fertilized. Today, considering the importance of environmental issues, increasing attention is being paid to different fertilizers and the methods of application of these organic and inorganic fertilizers. In Bangladesh, Hasanuzzaman et al. (2008) observed that aloe increases its number of leaves (+38%) and total leaf weight (+153%), with the application of 50% cowdung +50% soil, compared to the control (100% soil). García (2000) used one dose (4.6 t ha^−1^) of bio compost but with no clear effect on aloe growth. In Mexico, Murillo-Amador et al. (2007) showed that compost-based manure is effective and found that as the compost doses increased from 30 to 60 t ha^−1^, the leaves and gel yield increased (Figure 5D).

Nitrogen fertilizer may enhance both growth and yields because the nitrogen supply helps in the full expansion of the leaf, chlorophyll content, photosynthetic rates and subsequently increases the supply of carbohydrates to the plants (Khandelwal et al., 2009). The application of 150 kg N ha^−1^ resulted in an increased both number and size of leaves, and total yield (Bharadwaj, 2011).

Pedroza-Sandoval and Gómez-Lorence (2006) also reported the use of urea as an N source with one dose of 500 kg ha^−1^, and two applications per year.

The simultaneous application of nitrogen fertilizer and BA (1500 ppm), sprayed in the 17th week after planting, increases the number of leaves (Hazrati et al., 2012). The foliar application of amino acid had positive effects on the content of secondary metabolites, antioxidants, and antioxidant activity (Ardebili et al., 2012).

In order to make cultivation ever more competitive, the crop cycle should be reduced in order to obtain more rapid growth and improve quality. These results can be achieved with mycorrhizal inoculations. In many species, mycorrhizal symbiosis improves the transfer of nutritive elements to the roots, ensures greater plant growth, and stimulates the formation of a more dynamic root apparatus, making it more extensive and branched. In the literature, there are contrasting results regarding mycorrhizal applications in aloe (Tawaraya et al., 2007; Cardarelli et al., 2013; Sharma G. P. et al., 2013). This could be due to different cultivation environments, the species of fungi and the nitrogen fertilization regime. Colonization with *Glomus intraradices* and *G. mosseae* has a positive effect on the concentration of aloin and β polysaccharides (Cardarelli et al., 2013).

### Soilless cultivation

#### Container cultivation

The cultivation of aloe in containers has also become a standard technique in central and southern Europe. The rooted sucker is transplanted into a plastic pot, 2.5–3.5 L in volume and re-potted after 6–9 months into a 24 L container. The “container system” is derived from the association of plant, substrate, fertilizer and irrigation. It leads to a higher quality leaf production with reduced production time. The conventional substrate is made from a mixture of peat and other mineral components (sand or pumice or perlite) in v:v 1:1. Despite the expensive cost of peat, its ever-decreasing availability due to environmental restrictions and the possibility of applying local by-products has led to the use of alternative organic materials such as compost.

**Figure 6:**
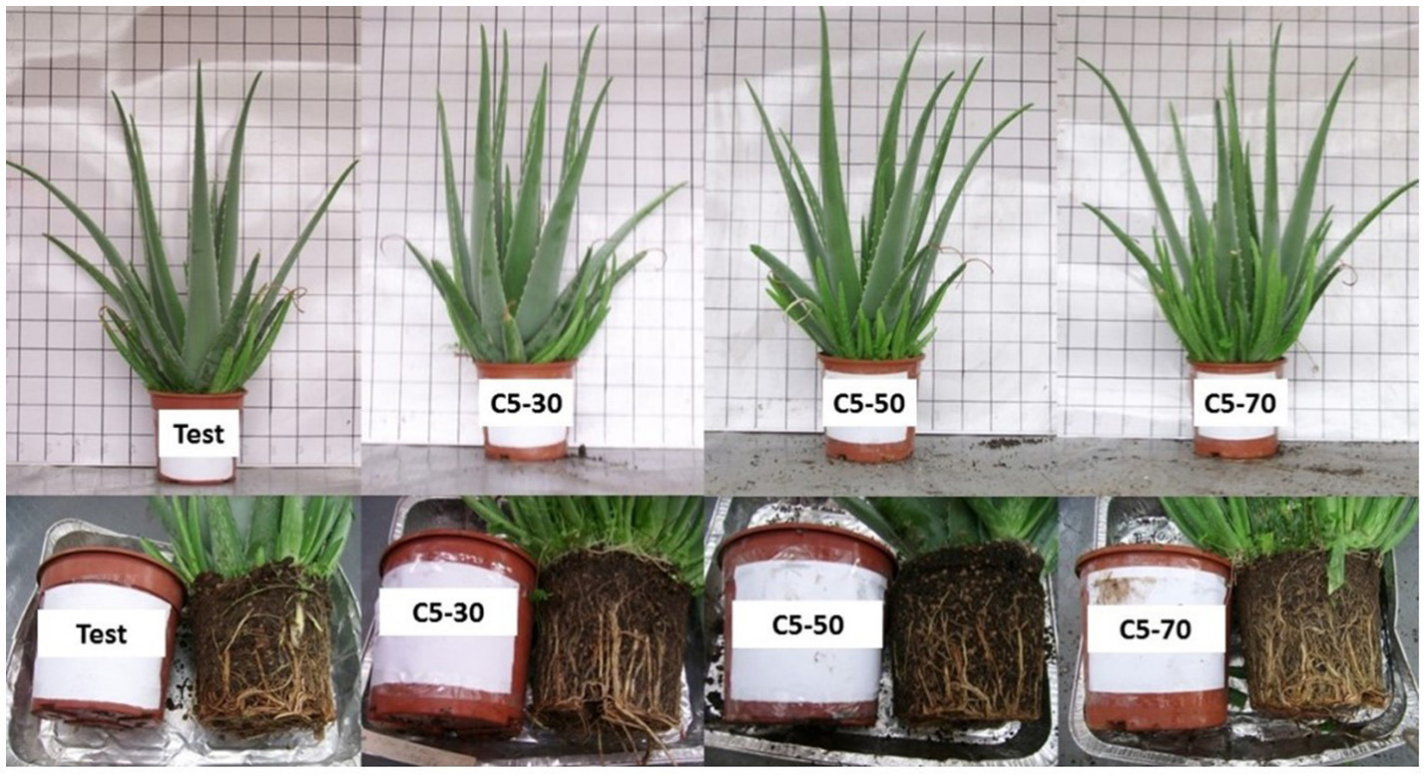
**Potted aloes: comparison between peat cultivation (test) and increasing rates of green compost in the second half-year of cultivation**. Photo taken by De Lucia.

Rea et al. (2008) evaluated the effect of the replacement (partially or totally) of peat, with increasing doses of green compost, derived from ornamental tree prunings. In the first half of cultivation, the replacement equaled the performances of both the plant height and diameter, and improved both leaf numbers and plant fresh weight (Table 4). In the second half, there were no significant differences between the substrates; plants did not show any phytotoxicity, and appeared disease free and with no weeds (Figure 6, Table 4).

**Table 4:** Effect of growing media containing rates of peat (Test) and green wastes compost (C5) on some morphological traits in first and second half-year cultivation (^*^).

	**1st half of cultivation**				**2nd half of cultivation**			
Growing medium (^**^)	**Height (cm)**	**Diameter (cm)**	**Leaves (n)**	**Fresh weight (g)**	**Height (cm)**	**Diameter (cm)**	**Leaves (n)**	**Fresh weight (g)**
**PLANT**								
Test	53a	56a	17b	2823b	61a	69a	21a	3935a
C5-30	53a	55a	20a	3433a	58a	69a	22a	3928a
C5-50	53a	60a	20a	3370a	57a	71a	22a	3943a
C5-70	55a	56a	21a	3565a	58a	69a	21a	3938a

*Means with the same letter within a column are not significantly different at P < 0.05*.

* *Data from Rea et al. (2008)*.

** *Test, 70% Sphagnum peat + 30% draining materials + 0%C5 green compost; C5-30, 40% Sphagnum peat + 30% draining materials + 30%C5 green compost; C5-50, 20% Sphagnum peat + 30% draining materials + 50%C5 green compost; C5-70, 0% Sphagnum peat + 30% draining materials + 70%C5 green compost*.

#### Hydroponic cultivation

One of the main constraints in hydroponic cultivation is to obtain a suitable nutrient solution with the highest yield and quality. However, the identification of optimal soilless practices such as nutrient solution preparation is critical in order to increase yield and quality. In *A. vera*, it may be possible to obtain higher yields of specific secondary metabolites or higher yields of target organs. Saliqehdar et al. (2013, 2014) developed a nutrient solution, using 9.7 meqL^−1^ NO_3_ and 5.8 meq·L^−1^ K, which produced the highest vegetative growth without negative effects on qualitative indices including aloin, total phenol, total antioxidative activity and element contents. Olfati et al. (2015) indicated that the NO_3_ supply combined with low quantities of ${\text{NH}}_{4}^{+}$ favor aloe growth.

## Abiotic stresses and bioactive molecules

In arid and semi-arid areas, sustainable agriculture, should aim to store water. Aloe is naturally adapted to dry conditions and high temperatures. In the Atacama Desert of Chile, which is one of the most arid regions of the world, the plants irrigated with 75% field capacity presented the best water use efficiency in terms of dry mass and amount of gel produced by a liter of supplied water. The osmotic adjustment is highlighted by an increase in the synthesis of proline, total soluble sugars as well as polymers of β- (2-6) kestotriose (Delatorre-Herrera et al., 2010).

Saline stress is a limiting factor for production and growth, and is becoming a serious problem in many parts of the world. Fortunately, *A. vera* grows well on saline soils of beach areas in tropical and subtropical xerophytic conditions (Rahi et al., 2013).

Several NaCl concentrations or sea water have been used to evaluate aloe salt tolerance. Jin et al. (2007) studied the physiological response to saline stress (EC: 23 dS m^−1^) which causes a decrease in the water content in the plant tissues as well as in total soluble sugars and glucose. Rahimi-Dehgolan et al. (2012) irrigated plants with fresh (EC control: 0.4 dS m^−1^) or saline lake water (EC: 3, 6, 9, 12, 15, 18, 21 dS m^−1^): irrigation with saline lake water up to 9 dS m^−1^, increased the concentration of glucose, xylose and mannose in the leaf gel.

Murillo-Amador et al. (2014) reported that Na and Cl ions accumulated in the cells of roots, stems, leaves and sprouts, thus showing higher Na and Cl contents in sprouts followed by leaves, stems and roots.

Murillo-Amador et al. (2015) found that using moderate salt stress (0, 30, 60, 90, and 120 mM NaCl) instead of typical high salt stress (>200 mM) offers a new perspective for directly studying the salinity-resistant mechanisms of desert plants and may lead to new salt-resistant cultivars. This prevents damage under NaCl treatments, thus increasing the agronomic value of aloe plants, making this species attractive for industrial production in arid or semiarid areas with moderate salinity. Murillo-Amador et al. (2015) also found that the relative water content of aloe plants shows values close to 98%, which demonstrates that the water content is not significantly affected in the leaves of plants growing under this regime of moderate salt stress. The accumulation of soluble carbohydrates as osmolytes in plants has been widely reported as a response to salinity or drought. Soluble sugars prevent protein dehydration because of turgor pressure (Crowe et al., 1990). Aloin concentration was found to increase with salt stress up to 15 dS m^−1^ whichdecreased at higher salinity concentrations, perhaps due to the low water content in leaves. Proline and PEP-case increased as salinity increased in both parenchyma and chlorenchyma of aloe under NaCl stress of 0, 30, 60, 90, and 120 mM, while total protein increased in parenchyma and decreased in chlorenchyma. The increase in protein, proline and PEP-case activity, as well as the absorption and accumulation of cations under moderate NaCl stress caused osmotic adjustment, which kept the plant healthy (Murillo-Amador et al., 2014).

Under NaCl stress, Xu et al. (2006) demonstrated that the addition of silicate (Si) to a nutrient solution significantly decreased the Na and Cl contents in aloe roots, stems and leaves, while K content and K/Na ratio increased significantly. The addition of Si enhanced the selectivity of roots to K absorption and the selectivity of stems and leaves to K translocation under NaCl stress.

## Harvest and post-harvesting

### Harvest

The older outer leaves are generally harvested, leaving the fresh and young leaves at the top. The plants can be removed manually or with the help of a tractor-drawn disc harrow or cultivator. New leaves grow from the center upward. Economic yields are obtained for 5 years, after which the Aloe needs replanting. Harvesting is a labor-intensive process. Only mature well developed leaves are collected, which are 60–80 cm in length and with a width at the base of around 8–10 cm. Typically, the outermost 3–4 leaves are harvested by pulling each leaf away from the plant stem. Leaves that show signs of tip necrosis should not be harvested, as these provide entry points for microbial contamination. If harvesting is done once a year, October-November is the best period. The second year gives the maximum yield, and for about 4–5 years a good yield can be harvested. Harvested leaves are carefully stacked and then transferred to a refrigeration or processing facility.

Employing good agronomic practices and with 20,000 plants ha^−1^ can provide two harvests per year (May and October). Each harvest involves the removal of, on average, four leaves and therefore approximately eight leaves per year^−1^ per plant^−1^. The mean weight of a mature fresh leaf reaches 500g, thus obtaining 4 kg plant^−1^, corresponding to approximately 80 ton ha^−1^ (Bassetti and Sala, 2003). With a commercial density of 10,000 plants ha^−1^ and similar edaphoclimatic conditions, the maximum productivity should reach 127 ton ha^−1^year^−1^, which is comparable to the value of 108 ton ha^−1^year^−1^ estimated for the production of green biomass in *Opuntia ficus indica* (Flores-Hernández et al., 2004). In a greenhouse experiment, Rodríguez-García et al. (2007) found a significant reduction in leaf biomass yield due to lower water availability. They estimated yields of 44.5–58.5 ton ha^−1^ year^−1^ for a density of 10,000 plants ha^−1^. In India, the average yield for organically grown aloe is about 12 ton ha^−1^ year^−1^ (Rajeswari et al., 2012).

### Post-harvesting

Post-harvest, aloe leaves behave differently depending on the region or country and according to the level of technology used in each aloe plantation. The gel or juice of Aloe leaves can be extracted from the complete leaf, i.e., including the yellow sap commonly called the “acibar” (Pedroza-Sandoval and Gómez-Lorence, 2006) or extracted in a way that is similar to how a fish is fileted, and the outside ring is removed, exposing the gel inside (Gage, 1996). The most precious commodity of the aloe plant is the juice, made from the clear gel stored inside the leaf. All reports of post-harvesting activities (Rodríguez-Canto, 1992; CONAZA, 1994; Gage, 1996; Granados-Sánchez and Castañeda-Pérez, 1998; Pedroza-Sandoval and Gómez-Lorence, 2006) refer to washing of the leaves using tap water alone or mixed with detergent, chloride, quaternary ammonium salts, sodium hypochlorite etc. Some processing plants also use manual or mechanized brushes to clean the leaves.

The main stages of the process of post-harvesting include: reception at processing plant, unloading, weighing, storage, washing 1, washing 2, removal of tips and bottoms, fileting, and removing the outside ring. Many aloe manufacturers use machines to filet the leaves, others believe that hand fileting produces the cleanest gel. After the leaves have been fileted, the inner jelly-like pulp is ground by a blender. The aloe is then reduced to a near-liquid state. Fresh aloe degrades when exposed to air, thus the gel must be treated to prevent oxidation and loss of the plant's vitamins, minerals, amino acids, and other ingredients.

## Gel processing

Most companies stabilize the gel by pasteurization and the addition of preservatives (sodium benzoate). However, processing techniques vary from company to company, region-to-region, and country to country, i.e., some companies use a fugitive sterilizing agent that sterilizes the product without any chemical reaction with aloe, and the agent is then completely removed, all without the need for any heat (Gage, 1996). Other preservation methods include keeping it under refrigeration or vacuum sealed in a can. After fileting, some companies dehydrate the leaves using hot air, after which they are ground by a blender and the dehydrated or granulated gel is packed to sell to other industries (Rodríguez-Canto, 1992).

One key issue with aloe juice is that it loses its nutritional quality after only 2 or 3 h. It thus needs to be stabilized by techniques such as hydrogen peroxide oxidation, ultraviolet rays in the presence of chemical catalysts, heat treatment (1.5–2 h), high temperatures (71–77°C) for 3 min, and quenching-pasteurization (Álvarez, 1987). After stabilization, the processed aloe gel may be filtered to remove the pulp, depending on the type of product the company makes. Some companies use activated carbon to discolor aloe juice so that the red color and initial bitterness of the juice are reduced. For cosmetics and some juice drinks, filtered aloe is preferred, but some people like unfiltered drinks because the pulp is a good source of fiber. After being filtered, some companies lyophilize the gel with high vacuum treatment at low temperatures to remove the moisture, thus obtaining a white-gray powder (freeze-dried gel). This aloe product has a high concentration since when 1 kg is rehydrated, 200 kg of filtered juice are obtained (Rodríguez-Canto, 1992; Pedroza-Sandoval and Gómez-Lorence, 2006).

Cosmetic quality gel is obtained after pasteurization, by centrifuging the juice. This gel is the main ingredient for shampoos, creams and body or face soaps (Pedroza-Sandoval and Gómez-Lorence, 2006). The other aloe gel category is food grade (liquid concentrate), which is the basis for certain dietetic foods (Gage, 1996) with preservatives to prevent deterioration by pollution.

Quality control is typically high in all aloe companies, and the product is tested for various properties such as color, viscosity, odor, Brix degrees, total solids, precipitable solids in methanol, sediment percentage, foreign matter, microbiological analysis such as plate full account, fungi and bacteria account, pathogens and coliforms, and pH balance (Pedroza-Sandoval and Gómez-Lorence, 2006).

## Conclusions

The pharmaceutical, cosmetics, and food industries are showing ever-increasing interest in, the expansion of aloe cultivation, as an alternative to traditional crops, even in arid and semi-arid environments.

Achieving high yield in leaves and gel quality requires an integrated approach from pre cultivation, to cultivation and post cultivation.

Further research is needed with regard to:

✓Increasing the efficiency of the protocols *in vitro* cultures via the callus;
✓Assessing the eco-physiological response on marginal lands with low quality water and low supply of organic matter to compost;
✓Improving, in soilless cultivation, the content of bioactive molecules in leaves;
✓Preserving and maintaining the bioactive chemical entities naturally present in the leaf in processing systems.

## Author contributions

Review conception: BD, GC. Wrote, edited and revised the paper: BD, GC, BM. Critical revision: BD, GC, BM. Approved the final version of the manuscript to be published: BD, GC, BM.

### Conflict of interest statement

The authors declare that the research was conducted in the absence of any commercial or financial relationships that could be construed as a potential conflict of interest.

## Abbreviations

2,4-D, 2,4: dichlorophenoxy acetic acid
AC: activated charcoal
ADS: Adenine sulfate
B5: Gamborg medium culture
BA: N6-benzyladenine
BAP: benzyl aminopurine
CAM: crassulacean acid metabolism
FYM: farm yard manure
IAA: Indole-3-acetic acid
IBA: Indole-3-butyric acid
Kin: (Kinetin) 6-furfurylaminopurine
MS: Murashige and Skoog medium culture
NAA: α-napthalene acetic acid
PGRs: plant growth regulators
PVP: Polyvinyl pyrolidine
SH: Sckenk and Hildebrant medium culture.
